# Navigating the Transition From Adolescence to Adulthood Among Young People With Severe Haemophilia: The Qualitative Phase of the TRANSHEMO Project

**DOI:** 10.1111/hae.70101

**Published:** 2025-09-11

**Authors:** Marie‐Anaïs Roques, Natacha Rosso‐Delsemme, Amandine Celli, Ngoc Anh Thu Nguyen, Martin Postzich, Sabine Castet, Yoann Huguenin, Annie Harroche, Anne Lienhart, Sandrine Meunier, Christine Biron‐Andréani, Florence Rousseau, Roseline d'Oiron, Yohann Repessé, Clémence Tabélé, Any Beltran Anzola, Thomas Sannié, Nicolas Giraud, Pascal Auquier, Hervé Chambost, Noémie Resseguier, The TRANSHEMO Study Group, Thémis Apostolidis, Laurent Ardillon, Karine Baumstarck, Sophie Bayart, Claire Berger, Marie‐Anne Bertrand, Carol Betsch, Annie Borel‐Derlon, Mohamed Boucekine, Thierry Calvez, Pierre Chamouni, Ségolène Claeyssens Donadel, Yannick Colle, Yesim Dargaud, Emmanuelle de Raucourt, Dominique Desprez, Céline Falaise, Alexandra Fournel, Birgit Frotscher, Valérie Gay, Fabienne Genre‐Volot, Jenny Goudemand, Yves Gruel, Benoît Guillet, Abel Hassoun, Audrey Hochart, Thierry Lambert, Aurélien Lebreton, Tanguy Leroy, Raphaël Marlu, Michèle Martin, Vanessa Milien, Fabrice Monpoux, Pierre Morange, Guillaume Mourey, Claude Negrier, Philippe Nguyen, Placide Nyombe, Caroline Oudot‐Challard, Brigitte Pan‐Petesch, Benoît Polack, Anne Rafowicz, Antoine Rauch, Delphine Rivaud, Pierre‐Simon Rohrlich, Pascale Schneider, Alexandra Spiegel, Cécile Stoven, Sophie Susen, Brigitte Tardy, Marc Trossaërt, Jean‐Baptiste Valentin, Stéphane Vanderbecken, Marie Viprey, Romain Voltzenlogel, Annelise Voyer‐Ebrard, Bénédicte Wibaut

**Affiliations:** ^1^ LPCPP / UR 3278 Laboratory of Clinical Psychology and Psychoanalysis Aix‐Marseille University Aix‐en‐Provence France; ^2^ Haemophilia Treatment Centre Children Hospital La Timone University Hospital of Marseille (AP‐HM) Marseille France; ^3^ Haemophilia Treatment Centre University Hospital of Nantes Nantes France; ^4^ CEReSS / UR 3279 Center for Studies and Research on Health Services and Quality of Life Aix Marseille University Marseille France; ^5^ Methodological Support Unit for Clinical and Epidemiological Research University Hospital of Marseille (AP‐HM) Marseille France; ^6^ Haemophilia Treatment Centre University Hospital of Bordeaux Bordeaux France; ^7^ Haemophilia Treatment Centre Hospital Necker, University Hospital of Paris (AP‐HP) Paris France; ^8^ Haemophilia Treatment Centre Hospital Louis Pradel University Hospital of Lyon (HCL) Bron France; ^9^ Haemophilia Treatment Centre University Hospital of Montpellier Montpellier France; ^10^ Haemophilia Treatment Centre Hospital Bicêtre University Hospital of Paris (AP‐HP) Paris France; ^11^ Haemophilia Treatment Centre University Hospital of Caen Caen France; ^12^ FranceCoag Network University Hospital of Marseille (AP‐HM) Marseille France; ^13^ French Association for people with Haemophilia (AFH) Paris France; ^14^ LPS / UR 849, Social Psychology Laboratory Aix‐Marseille University Aix‐en‐Provence France; ^15^ Haemophilia Treatment Centre University Hospital of Tours Tours France; ^16^ Haemophilia Treatment Centre University Hospital of Rennes Rennes France; ^17^ Haemophilia Treatment Centre University Hospital of Saint‐Etienne Saint‐Etienne France; ^18^ Haemophilia Treatment Centre University Hospital of Besançon Besançon France; ^19^ UMR‐S 1136, Pierre Louis Institute of Epidemiology and Public Health Sorbonne University, INSERM Paris France; ^20^ Haemophilia Treatment Centre University Hospital of Rouen Rouen France; ^21^ Haemophilia Treatment Centre University Hospital of Toulouse Toulouse France; ^22^ Haemophilia Treatment Centre Hospital of Versailles Versailles France; ^23^ Haemophilia Treatment Centre University Regional Hospital of Strasbourg Strasbourg France; ^24^ Haemophilia Treatment Centre University Hospital of Nancy Nancy France; ^25^ Haemophilia Treatment Centre Hospital of Chambéry Chambéry France; ^26^ Haemophilia Treatment Centre University Hospital of Dijon Dijon France; ^27^ Haemophilia Treatment Centre University Regional Hospital of Lille Lille France; ^28^ Haemophilia Treatment Centre Hospital of Simone Veil d'Eaubonne Montmorency France; ^29^ Haemophilia Treatment Centre University Hospital of Clermont‐Ferrand Clermont‐Ferrand France; ^30^ Haemophilia Treatment Centre University Hospital of Grenoble Grenoble France; ^31^ Haemophilia Treatment Centre University Hospital of Nice Nice France; ^32^ Laboratory of Haematology, Hospital La Timone University Hospital of Marseille (AP‐HM) Marseille France; ^33^ Haemophilia Treatment Centre University Hospital of Reims Reims France; ^34^ Haemophilia Treatment Centre University Hospital of Reunion Reunion Island France; ^35^ Haemophilia Treatment Centre University Hospital of Limoges Limoges France; ^36^ Haemophilia Treatment Centre University Hospital of Brest Brest France; ^37^ Haemophilia Treatment Centre University Hospital of Amiens Amiens France

**Keywords:** adherence, adolescents, haemophilia, quality of life, transition, young adults

## Abstract

**Introduction:**

Haemophilia causes spontaneous or prolonged bleeding due to a deficiency in clotting factor VIII (haemophilia A) or IX (haemophilia B). Although substitutive therapies and regular follow‐up can prevent severe haemorrhagic events, adherence to treatment remains a challenge. Transitioning from adolescence to adulthood and from paediatric to adult care is particularly complex for young people with severe haemophilia (PwSH), as it involves gaining autonomy in health management.

**Objectives:**

This study aimed to explore factors influencing the success of the transition process in young PwSH, with a focus on adherence to healthcare.

**Methods:**

This qualitative study was part of the mixed‐methods TRANSHEMO project. Participants were selected from the quantitative phase of the TRANSHEMO project based on two criteria: adolescents/young adults and adherent/nonadherent to healthcare. Interviews were conducted via video conferencing, transcribed, and thematically analysed to identify key themes affecting the transition process.

**Results:**

Twenty‐two interviews were conducted. Four major themes emerged as critical to transition success: (1) Care factors [continuity of care, treatment rituals, and evolving therapies]; (2) Family and social factors [support from family, friends, peers, and overprotection]; (3) Personal factors [understanding haemophilia, risk management, optimism, and coping strategies]; and (4) Autonomy [secondary benefits, independence, proactivity in disease management, and accompaniment by caregivers].

**Conclusion:**

Based on the enlightened determinants, supportive strategies and patient education programs should focus on the development of autonomy, personal factors such as acquisition and application of health literacy in haemophilia care, and family factors such as support from family.

## 1 Introduction

Haemophilia is a rare genetic disorder characterized by spontaneous or prolonged bleeding due to a deficiency or absence of clotting factor VIII (haemophilia A) or IX (haemophilia B) and transmitted via X‐linked recessive inheritance, thus affecting mainly males [1]. People with severe haemophilia (PwSH) may present serious haemorrhagic events (e.g., intracranial haemorrhage, haemarthrosis). It is possible to avoid these events by haemophilia care, such as substitutive therapies and lifelong regular clinical follow‐up, whose major issue is adherence. Severe haemophilia, like other severe chronic conditions, can significantly impact patients' lives from birth to adulthood, giving rise to specific concerns at different stages of life [2]. Patients’ developmental process requires them to progress from being entirely dependent on caregivers to becoming independent and gaining autonomy in adulthood.

The process of transitioning to adult life can be complex for young people with a chronic disease because it involves navigating the transition between adolescence and adulthood, and transferring from a paediatric to an adult care system [3, 4]. A successful transition requires the transfer of responsibility for managing their health from parents to children, mobilizing their experiential knowledge, acquiring new knowledge and skills necessary for autonomy, and accepting their new role as an adult [5, 6, 7]. A difficult transition can lead to a significant decrease in adherence as well as a deterioration in the patient's overall health and quality of life (QoL) [8, 9].

According to the theoretical Social‐ecological Model of Adolescents and Young Adults' Readiness for Transition (SMART) [10], several factors affect the success of the transition process for young people with chronic disease [11, 12, 13, 14, 15, 16], such as factors associated with young people themselves, with their parents, and with healthcare providers [10, 17, 18, 19, 20, 21, 22].

In the specific context of haemophilia, qualitative research has examined the transition of young people [23, 24, 25, 26, 27]. However, no such studies have been conducted in the framework of a mixed methods design to address the issue of transition in young PwSH in a global way.

The general objective of the mixed methods TRANSHEMO project [5, 28] was to explore the transition to adulthood of young PwSH in a global way. The present study, which is the qualitative phase of the project, aimed to explore participants’ views in more depth (patients were selected from the quantitative phase) to explain and refine the understanding of the quantitative phase.

## 2 Materials and Methods

### 2.1 Population

Participants included in the quantitative research met the following inclusion criteria: adolescents aged 14–17 years and young adults (YAs) aged 20–29 years, PwSH A or B, included in the FranceCoag registry, and followed in one of the 29 participating haemophilia treatment centres (HTCs).

Participants included in the qualitative phase were selected from the quantitative phase according to four types defined by two characteristics: age (adolescents vs. YAs) and adherence to healthcare (adherent vs. nonadherent), this adherence being considered as a marker of the success of the transition to adulthood [5].

### 2.2 Methods

A researcher conducted the interviews between December 2020 and June 2021 via the Zoom online conferencing platform due to the COVID‐19 pandemic. The interviews lasted 45 min to 1 h and followed a predetermined open‐ended interview guide that allowed participants to explore specific responses. The guide encouraged participants to discuss their understanding of the transition to adulthood, their expectations for their personal and professional lives, their plans for managing their health, their fears about entering adulthood, their difficulties in acquiring autonomy, and the barriers and facilitators they encountered during their transition process.

### 2.3 Sample Size

The number of participants was determined using a data saturation method.

### 2.4 Ethics

The study was approved by the French Ethical Research Committee (reference number 16.065), by the French National Agency for the Safety of Medicines and Health Products (reference number ID RCB: 2016‐A01034‐47), and by the French Data Protection Authority (reference number DR‐2018‐060), whose principles are in line with those of the General Data Protection Regulation. The protocol was registered at ClinicalTrials.gov (NCT02866526). To participate in the quantitative phase of this study, written consent was obtained from the YAs and the parents (or legal representatives) of the adolescents. Additional consent for voice recording was obtained from participants who agreed to participate in the qualitative phase of the study.

### 2.5 Analysis

The interviews were analysed using thematic analysis. A realist approach was adopted, reporting the experiences, meanings, and realities of the participants. Each theme mentioned at least once by the participants was considered, because the number of occurrences is not necessarily a reliable indicator of a theme's importance [29]. The steps outlined by Braun and Clarke [29] were followed, including familiarizing the researchers with the data, generating initial codes, searching for themes, reviewing themes, defining and naming themes, and producing the final report.

## 3 Results

### 3.1 Participants

We recruited 22 French‐speaking participants from the quantitative phase of the TRANSHEMO project [5, 28]. Their main characteristics are presented in Table 1.

**TABLE 1:** Characteristics of adolescents and young adults.

	Adolescents (*n* = 10)		Young adults (*n* = 12)	
	Adherent people (*n* = 7) *n*/mean (%/SD)	Non‐adherent people (*n* = 3) *n*/mean (%/SD)	Adherent people (*n* = 6) *n*/mean (%/SD)	Non‐adherent people (*n* = 6) *n*/mean (%/SD)
Sociodemographic characteristics				
Age (years)	14.9 (1.1)	15.0 (1.0)	25.0 (1.8)	22.3 (2.1)
Sex				
Male	7 (100)	3 (100)	6 (100)	6 (100)
Female	0	0	0	0
Highest level of education attained				
Junior school or vocational qualification	3 (42.9)	1 (33.3)	0	0
High school	4 (57.1)	2 (66.7)	1 (20.0)	3 (60.0)
Higher education	0	0	4 (80.0)	2 (40.0)
*Not available*	*0*	*0*	*1*	*1*
Academic difficulty (at least one grade repetition) *(yes)*	0	0	3 (50.0)	2 (33.3)
Socioeconomic status of the family				
Low or medium	2 (28.6)	3 (100)	4 (66.7)	3 (50.0)
High	5 (71.4)	0	2 (33.3)	3 (50.0)
Member of AFH *(yes)*	4 (57.1)	0	2 (33.3)	2 (33.3)
Clinical characteristics				
Type of haemophilia				
Haemophilia A	5 (71.4)	3 (100)	5 (83.3)	4 (66.7)
Haemophilia B	2 (28.6)	0	1 (16.7)	2 (33.3)
At least one comorbidity/complication *(yes)*	0	0	1 (16.7)	2 (33.3)
HIV infection *(yes)*	0	0	0	0
HBV infection *(yes)*	0	0	1 (16.7)	0
HCV infection *(yes)*	0	0	0	0
History of intracranial haemorrhage *(yes)*	0	0	0	0
History of major orthopaedic interventions *(yes)*	0	0	0	0
Current treatment regimen				
On‐demand treatment	0	0	3 (50)	3 (50)
Prophylactic treatment	7 (100)	3 (100)	3 (50)	3 (50)
Psycho‐behavioural factors				
Family functioning (score of FAD)	1.8 (0.6)	2.1 (0.7)	1.8 (0.3)	1.4 (0.6)
Participation in therapeutic education activities *(yes)*	5 (50)	0	2 (33.3)	3 (50.0)
Quality of life				
Physical health (PCS)	51.9 (9.8)	50.2 (3.8)	44.6 (14.1)	50.6 (11.8)
Mental health (MCS)	49.6 (12.0)	49.6 (12.0)	49.9 (5.9)	46.2 (12.7)
Haemophilia‐related quality of life (Haemo‐QoL)	21.4 (19.4)	24.0 (14.1)	41.7 (27.3)	30.7 (30.4)
Coping strategies (scores of Brief COPE)				
Social support	3.7 (1.0)	3.8 (1.3)	4.6 (0.6)	4.2 (1.5)
Problem solving	5.6 (1.5)	4.7 (0.8)	5.6 (1.1)	6.2 (1.2)
Avoidance	3.3 (0.7)	3.5 (1.0)	3.4 (0.4)	3.6 (0.5)
Positive thinking	5.4 (1.3)	4.7 (1.5)	5.3 (1.0)	5.6 (1.2)
Autonomy (scores of Noom)				
Attitudinal autonomy	18.9 (2.3)	20.0 (3.6)	16.8 (4.4)	17.8 (7.0)
Emotional autonomy	18.4 (3.2)	19.0 (1.0)	16.2 (2.7)	18.7 (3.9)
Functional autonomy	19.7 (2.7)	17.0 (2.6)	18.8 (4.6)	16.3 (4.7)
Time perspective (scores of ZTPI)				
Past negative	2.2 (0.8)	2.4 (1.1)	3.0 (0.4)	2.7 (1.1)
Future	3.3 (0.5)	3.3 (0.3)	3.7 (0.3)	3.7 (0.4)
Organizational (HTC‐related) factors				
Paediatrician in the medical team of the HTC *(yes)*	7 (100)	3 (100)	6 (100)	4 (66.7)
Type of healthcare transition in the HTC				
Same medical team for adolescents and adults	6 (85.7)	1 (33.3)	4 (66.7)	5 (83.3)
Two different medical teams (paediatric/adult)	1 (14.3)	2 (66.7)	2 (33.3)	1 (16.7)
Set‐up of therapeutic education activities about transition by the HTC *(yes)*	0	0	0	2 (33.3)

Abbreviations: AFH, French Association for people with Haemophilia; Brief COPE, Brief Coping Orientation to Problems Experienced; FAD, Family Assessment Device; HBV, hepatitis B virus; HCV, hepatitis C virus; HIV, human immunodeficiency virus; HTC, haemophilia treatment centre; MCS, mental component score measured by SF‐12 (Short form 12 Health Survey); PCS, physical component score measured by SF‐12 (Short form 12 Health Survey); ZTPI, Zimbardo Time Perspective Inventory.

### 3.2 Results of the Research

The results of the thematic analysis are presented in Table 2. Our analysis revealed four major themes that played a crucial role in facilitating or limiting the success of the transition: (1) care factors, (2) family and social factors, (3) personal and personality factors, and (4) autonomy.

**TABLE 2:** Thematic analysis.

Themes	Subthemes	Verbatim	Adherent Adults *n* = 6	Adherent Adolescents *n* = 7	Non‐adherent Adults *n* = 6	Non‐adherent Adolescents *n* = 3
**Care factors**	*Follow‐up by the same medical team*	*“I go there once a year for a routine check‐up. Since I started to be taken care of in this HTC, there were two or three doctors, but I haven't really felt the change because the doctors are very precise. They read my medical file as it's in the same HTC. They always knew my medical history and the fact that they are specialized in haematology and haemophilia, it gives confidence. I know that I can ask my questions to a specialist.” (Adh‐Adu 4)*	4	7	5	3
	*Ritualization of treatment*	"*Right now, I organize my injection on a fixed day, every Thursday morning and every Sunday night. I know that I have to do it at that time, so I never forget. It's like brushing my teeth.” (Adh‐Ado 3)*	2	4	0	0
	*Evolution of treatments and comfort care*	*“Now, with my new treatment, I don't have the same concern. My life changed radically; I couldn't have dreamed better. Yesterday, the doctors told me that I could increase the doses, so I only have one injection every 15 days. It gets better and better. I feel freer.” (Adh‐Ado 2)*	3	5	2	0
**Family and social factors**	*Support and protection from parents in childhood*	*“From the beginning, my parents have been invested in my care and in my treatments. We used to go to the hospital often and they would drive back and forth every time for my appointments. Having them around helped a lot. My sister has also been present for me all along.”* (Adh‐Ado 2)	6	7	3	3
	*Support and caring from friends*	*“I have very good friends, and I know that… I mean we have never really talked about it, but I know that if I don't feel good, they call me and we can discuss. They are supportive.” (Adh‐Ado 1)*	6	5	2	3
	*Identification with other people with haemophilia*	*“It helped me to meet other people with haemophilia…and I also think that it helped my parents.” (Adh‐Ado 6)*	4	6	4	3
	*Concern and overprotection from relatives*	*“My parents were very protective… probably too much. I would have loved for them to give me more freedom and space, but today I understand that I was lucky. When you're young, you want your independence. You don't want to be reminded about the disease… But now I understand that they did it to protect me.” (Adh‐Adu 6)* “*Well… now they are less protective because I'm older but when I was younger it was complicated for me. My mom was very protective, putting a shell around me… but at the same time she took care of everything, so I had less to think about. She still takes care of a lot of things today.” (N‐Adh‐Ado 1)*	4	3	2	2
**Personal and personality factors**	*Carefulness and farsightedness*	*“I've never taken too many risks; I've always been careful. Early on I realized that I need to be mature, and I can have fun, but I need to be careful. You need to strike a balance, because there is no point in taking risks and be damaged at the end.” (Adh‐Adu 1)*	5	5	3	3
	*Good knowledge of haemophilia, one's own body and limitations*	“*I've always done lots of activities. I've never felt limited. Of course, I don't do rugby, but it's alright. Rowing and sailing are good. You can do it with haemophilia.” (Adh‐Ado 6)*	3	6	3	3
	*Optimism*	*“Today, I don't feel bothered because I think it's already good that we have a treatment. For some diseases, people don't have treatment. Plus, they evolve in a good way, so I really feel optimistic.” (Adh‐Ado 2)*	6	7	4	3
	*Minimization and distancing of the disease*	*“I've never wanted to be autonomous; I've always wanted my mom to keep on taking care of me. I feel it's another barrier between me and the disease.” (N‐Adh‐Adu 2)*	2	0	6	2
**Autonomy**	*Secondary benefits of the disease*	*“I like that my mom is still involved in my care. I don't have to bear the burden all alone, and then I get to have special attention as well.” (N‐Adh‐Adu 2)*	2	2	4	3
	*Autonomy in the disease concomitant with independence in life*	*“I became autonomous when I left my parents’ house to go study in another city. I already know how to do my injection, but leaving my family made me fully independent.” (Adh‐Adu 2)*	4	4	4	3
	*Autonomy associated with a desire to be proactive in life and with acceptance of the illness*	*“My transition toward autonomy is pretty easy because it's way better and handier to be independent in the treatment. More generally in life, you can be freer; you can make your own choices, live alone, go traveling.” (Adh‐Ado 2)*	5	6	3	3
	*Accompaniment toward autonomy by the HTC*	*“I used to get on well with my nurse. When I was in the hospital, she asked me if I wanted to learn how to inject myself. My nurse gave me a fake plastic arm to train, and then I tried on her arm and finally I tried on mine, and it worked. It was good because then I could do it myself and go on vacations with friends without care about who would do the injections.” (Adh‐Adu 6)*	6	5	1	3

#### 3.2.1 Care Factors

**Being followed by the same medical team** appeared to be associated with a greater sense of confidence and support for all four groups of participants. According to most participants, this factor appeared to have a positive impact on the transition from adolescence to adulthood.

**Having routinized habits** such as performing injections at the same time of day or following a set sequence, which participants described as treatment rituals, appeared to facilitate the transition for adherent participants, because it helped them remember and manage their chronic disease better. One participant even compared it to a daily hygiene routine, saying: ‘It's like brushing my teeth’ (Adh‐Ado 3, Table 2). Participants who did not have rituals seemed to be less involved in their care process and, as a result, less adherent to treatments.

Participants perceived that the **therapeutic innovation** was associated with a higher QoL and more freedom in daily life. For most adherent participants, this factor appeared to facilitate the transition, as they maintained hope and benefited from the latest treatments.

#### 3.2.2 Family and Social Factors

The participants identified **the overall support of their family,** including involvement in care, as a helpful factor in the transition process, specifically in the normalization of their illness.

The participants also mentioned the positive impact of **social support,** such as friends with a good understanding of their condition.

**Receiving social support from other people with the same condition** appeared to be an important factor in facilitating the transition to adulthood. This support allowed the participants to find role models and work toward accepting their condition, whether this support came from family members, members of the French Association for people with Haemophilia, expert patients, or people from the same HTC.

The seriousness of the disease could lead to **overprotection and overcautiousness** from family members, which had both positive and negative effects on the transition process. Adherent participants often attributed their parents' behaviour to their precarious medical condition. For nonadherent participants, the overprotection and limitations imposed by their families could lead to feelings of helplessness and little involvement in their care.

#### 3.2.3 Personal and Personality Factors

***Being careful about risks and farsighted about the disease*** seemed to be important personality factors that facilitated the transition process. These factors seemed to be linked to respect for medical recommendations and advice.

**Having a good understanding of haemophilia, one's own body and limitations** seemed to be an important factor facilitating the transition process. This factor was associated with the ability to make their own choices in life (such as school, profession and hobbies), while considering their health condition and knowing what they could and could not do.

The **ability to maintain optimism about the consequences of haemophilia** seemed to be an important factor linked to a successful transition. This factor was also associated with the participants' ability to remain optimistic about the future, particularly with regard to medical progress and the evolution of treatments over time, as well as their perception of themselves as they aged.

The **ability to use coping strategies that involved minimizing or distancing** oneself from the condition appeared to be important among nonadherent participants. Specifically, they seemed not to fully recognize the seriousness of the illness and took more risks to test their limits. Most participants in this group also showed less interest in discussing haemophilia and expressed a sense of indifference and passivity toward their care management.

#### 3.2.4 Autonomy

***Having secondary benefits from the disease*** appeared to be a limiting factor in the transition process. This factor was mostly cited by nonadherent participants. Some of them felt comfortable not managing their treatment autonomously, which reduced their mental load.

**Being independent in life and leaving the parental home** seemed to be facilitators in the transition process. Autonomy in disease management is usually concomitant with autonomy in life.

**Having the desire to be proactive in life** facilitated the transition to adulthood and fostered the process of achieving autonomy. Being proactive appeared to be linked with acceptance of the disease. Adherent participants wanted to make deliberate choices about their condition and wanted to feel free in managing their treatment.

**Being accompanied toward autonomy by the HTC** appeared to be an important factor that facilitated the transition process. Increased autonomy seemed to be facilitated by the support and availability of the HTC's medical and paramedical team starting in early adolescence and continuing until the achievement of full autonomy. Several participants described how nurses or other healthcare professionals played a key role in gradually helping them become independent in managing their treatment. This included learning to self‐inject through hands‐on practice and receiving encouragement and reassurance. This supportive process enabled participants to gain confidence and manage their condition more freely.

## 4 Discussion

The period of transition from adolescence to adulthood is a critical time in terms of healthcare adherence for people with chronic diseases. This study compared the experiences of adolescents and YAs with severe haemophilia by contrasting their narratives according to their adherent/non‐adherent status. While adherence was used as a criterion to define participant profiles, our analysis aimed to investigate a broader, multidimensional understanding of transition success, encompassing psychosocial and structural dimensions in addition to adherence. Adolescents shared their perspectives and preparations for the transition, while YAs described their own experiences. Several factors, grouped across four dimensions, either hindered or facilitated transition success and adherence.

### 4.1 Factors Limiting Adherence to Healthcare and Transition Success

#### 4.1.1 Care Factors

**Perceiving treatments as a constraint** was a barrier to the successful transition. Patients who express difficulty in accepting haemophilia see their treatment as a constant reminder of their illness [30, 31]. Thus, these patients may consciously choose to skip their treatment. Furthermore, a lack of routine or ritualization limited adherence to healthcare. Nonadherent participants had difficulties in organizing their treatment injections, which resulted in forgetfulness and delays. This finding was supported by a previous study that highlighted the link between self‐management skills (planning injections, management of the illness, etc.) and adherence to healthcare as a lens rather than a sufficient measure of transition success [32].

#### 4.1.2 Family and Social Factors and Autonomy

**Overprotection and overcautiousness from parents** could limit transition success. For nonadherent participants, it appeared to be related to their difficulty in being involved in their own care. In addition, nonadherent participants cited the **secondary benefits they received**, such as special attention from their relatives and friends and comfort from not being autonomous in their care. This situation creates a vicious circle in which young PwSH do not take an active role in their healthcare due to the overinvolvement of their parents, who in turn become even more involved because of their child's lack of involvement. Previous research supports these findings; an educational style marked by overprotection was shown to be associated with lower adherence among children with chronic illnesses [30], highlighting the importance of finding the right balance between prevention and overprotection.

#### 4.1.3 Personal and Personality Factors

**Minimizing the impact of the illness and using distancing strategies** limited transition success. Some patients may be uninformed or uninterested in their illness and its potential consequences, leading them to ignore their health condition and make risky choices [30]. Others may struggle to emotionally accept their illness, which may lead to passivity in managing their own care and a lack of adherence to healthcare [33].

### 4.2 Factors Facilitating Adherence to Healthcare and Transition Success

#### 4.2.1 Care Factors

**Being followed by the same medical team** throughout the entire care pathway facilitated transition success because it increased the sense of confidence and support. Some studies have shown a link between being followed by the same medical team, having a stronger relationship with healthcare providers and better adherence to healthcare [30, 34]. In situations where clinical follow‐up could not be continued with the same medical team, participants mentioned the importance of communication between the two medical teams because transfers need to be prepared in advance for a bond of trust with new healthcare providers.

**Having a ritual and maintaining a regular schedule** facilitated transition success. Adherent participants described this routine as involving practical skills in medication preparation and infusion. Having a ritual could reduce stress and the risk of forgetting to take medication [34]. Previous research linked the ability to establish a treatment routine with improved forecasting of treatment needs, since people become more proactive and fully integrate their care into their daily lives [4]. **The perception of treatment benefits** facilitated adherence to healthcare [30]. Adherent participants expressed optimism about the future and the greater freedom that improved treatments allowed.

#### 4.2.2 Family and Social Factors

**Receiving parental support (including overprotection and overcautiousness), support from friends, and support from other PwSH through the HTC or associations** facilitated transition success. Adherent participants reported their parents' involvement in managing their treatment and care, as well as normalizing and reassuring them about their condition. This support enabled young PwSH to become more autonomous as they grew up and helped them develop the capacity to manage their health condition, self‐care, and self‐efficacy. Receiving social support from friends during the transition helped individuals feel “normal” and created a sense of belonging despite their condition. Peer support allowed access to other experiences and helped individuals feel “normal”, providing hope for the future. This support could take different forms, including participating in patient education programs contributed by expert patients [35], sharing practical advice [33], feelings, and personal experiences, leading to the feeling of being understood [34]. While peer support was largely viewed positively, some young PwSH preferred to distance themselves from their diagnosis, as part of a strategy to minimize the psychological burden of illness. This variation in responses highlights the need for personalized approaches to transition support, as not all individuals wish to identify with a patient group or revisit their illness outside of clinical care.

#### 4.2.3 Personal and Personality Factors

**Having a good understanding of haemophilia, one's own body, and its limitations** facilitated adherence and transition success. This involves understanding why bleeding occurs, when and how to take care of it, and who to turn to for support [36]. It allows young PwSH to better understand the importance of treatment, which could lead to appropriate health decision‐making. Furthermore, most adherent participants paid attention to their bodily sensations and symptoms, which enabled them to better manage their health [33]. It also made them more **careful about risks** and about integrating them into their daily lives and life choices. Acceptance of the disease led PwSH to adapt their life goals to their medical condition and make appropriate choices, helping to enhance resilience and **maintain optimism**.

#### 4.2.4 Autonomy

**The progressive acquisition of autonomy,** which allows young PwSH to become more **proactive** in managing their disease, facilitates transition success. People who gradually **accept** their chronic illness and learn to manage it over time develop their personal image in the process [37].

**Being accompanied by the HTC toward the autonomy of one's own care** facilitated transition success. Research has highlighted the positive role of medical teams in achieving autonomy, encouraging involvement in medical appointments [38], providing positive feedback, and discussing difficult times from the beginning of adolescence because the self‐management process is gradual [20].

### 4.3 Limitations and Strengths

First, adherence to healthcare as a criterion for a successful transition is still debatable. Nevertheless, adherence to healthcare is in the top five of healthcare transition outcomes validated by an interdisciplinary group of medical and psychosocial professionals [39]. Moreover, adherence to healthcare, which has often been assessed only by adherence to prophylaxis, was assessed more completely in our study by including seven subcriteria of adherence. In addition, transition success may encompass aspects such as QoL, autonomy, and self‐efficacy. The quantitative phase of our project [28] analysed the associations between psychosocial aspects (autonomy, mental health concerns, haemophilia‐specific QoL, social support) and adherence to healthcare, while the findings of the qualitative phase on lived experience emphasize the need for deeper studying perspectives on therapeutic innovations, relational or psychological vulnerabilities, preventive and reactive behaviours in acute bleed management of young PwSH to enhance their adherence to healthcare and QoL.

Second, some major therapeutic innovations, such as Emicizumab and gene therapy, have been recently developed and are now widely available, which was not the case at the time of the TRANSHEMO project. Adherence to healthcare for young PwSH may have evolved thanks to these therapeutic advances. It will be necessary to reassess this adherence in future studies.

Third, all interviews were conducted in French with participants living in France. This could limit the generalizability of the findings to other sociocultural contexts. While the identified themes, such as the importance of family dynamics, treatment ritualization, or autonomy, may resonate widely, cultural and organizational differences may affect their relevance. The decision to include only French‐speaking people was pragmatic, aiming to ensure the depth and nuance of qualitative data. However, this may have excluded migrants or non‐native French‐speaking individuals, who could face unique challenges related to cultural and linguistic integration. Future cross‐cultural studies could explore how social and cultural norms shape the transition process in PwSH.

Lastly, interviews were conducted remotely via Zoom during the COVID‐19 pandemic. Social isolation may have influenced participants’ perceptions and narratives. However, we did not observe signs of discomfort or constraint. On the contrary, many participants shared emotionally rich and personal stories, suggesting that the remote format did not hinder openness. Nonetheless, we acknowledge this potential limitation.

Despite these limitations, the study offers a rich and nuanced understanding of the transition process within a specific healthcare system and time frame.

### 4.4 Clinical Perspectives

The findings of this study can support practical clinical perspectives in both paediatric and adult care. To better support young PwSH during their transition to adulthood, we propose three complementary strategies.

First, healthcare providers need to provide intensified care support at transition points and take an active part in relaying information from paediatric to adult care centres and from one healthcare provider to another, when patients change doctors or hospital services. Improving communication between children's and adults’ hospitals and between healthcare providers can also help young PwSH and their families become more aware of the transition of care and ensure the continuity of care. Adults’ hospitals may offer a welcoming and competent environment, supported by stable professionals and trust‐building strategies. Early preparation and collaborative transfer planning can ease patient adaptation. In this context, paediatric teams should implement structured transition programs focusing on self‐injection and fostering broader healthcare autonomy, including appointment scheduling and treatment adaptation. Open discussions around identity and illness should be encouraged for emotional readiness.

Second, parents and families need more resources and information on transition to support young PwSH and to manage their own responsibility shift. This could help young PwSH gain autonomy. Structured parental involvement can enable progressive self‐management in adolescents. Families should gradually adjust their involvement and support to prevent overprotection and foster independence in disease management.

Third, young PwSH need more education before transitioning to gain a better understanding of their health condition, as well as to acquire health literacy in haemophilia care [40]. Peer interactions between PwSH can be a valuable source of reassurance, helping to communicate with people who share the same experience, offer emotional support, feel less isolated and exchange practical knowledge to facilitate the transition to adulthood.

By viewing transition as a multidimensional process, rather than as a binary success/failure defined by adherence to prophylaxis alone, care models can more effectively promote long‐term well‐being and QoL for young PwSH.

## Author Contributions

Marie‐Anaïs ROQUES, Natacha ROSSO‐DELSEMME, Amandine CELLI, Noémie RESSEGUIER, Pascal AUQUIER, Hervé CHAMBOST, Tanguy LEROY, Ngoc Anh Thu NGUYEN, Any BELTRAN ANZOLA, Nicolas GIRAUD, Thomas SANNIE, Thémis APOSTOLIDIS, Thierry CALVEZ and Pierre‐Emannuel MORANGE conceived the design of this study. The investigators of the French Haemophilia Treatment Centres (Laurent ARDILLON, Sophie BAYART, Claire BERGER, Marie‐Anne BERTRAND, Christine BIRON‐ANDREANI, Annie BOREL‐DERLON, Sabine CASTET, Amandine CELLI, Hervé CHAMBOST, Pierre CHAMOUNI, Ségolène CLAEYSSENS DONADEL, Yesim DARGAUD, Emmanuelle DE RAUCOURT, Dominique DESPREZ, Roseline D'OIRON, Céline FALAISE, Alexandra FOURNEL, Birgit FROTSCHER, Valérie GAY, Fabienne GENRE‐VOLOT, Jenny GOUDEMAND, Yves GRUEL, Benoît GUILLET, Annie HARROCHE, Abel HASSOUN, Audrey HOCHART, Yoann HUGUENIN, Thierry LAMBERT, Aurélien LEBRETON, Anne LIENHART, Raphaël MARLU, Michèle MARTIN, Sandrine MEUNIER, Vanessa MILIEN, Fabrice MONPOUX, Guillaume MOUREY, Claude NEGRIER, Philippe NGUYEN, Placide NYOMBE, Caroline OUDOT‐CHALLARD, Brigitte PAN‐PETESCH, Benoît POLACK, Anne RAFOWICZ, Antoine RAUCH, Yohann REPESSE, Delphine RIVAUD, Pierre‐Simon ROHRLICH, Florence ROUSSEAU, Pascale SCHNEIDER, Alexandra SPIEGEL, Cécile STOVEN, Sophie SUSEN, Brigitte TARDY, Marc TROSSAËRT, Jean‐Baptiste VALENTIN, Stéphane VANDERBECKEN, Annelise VOYER‐EBRARD, Bénédicte WIBAUT) enrolled participants. The members of the French Association for people with Haemophilia (BETSCH Carol, COLLE Yannick, GIRAUD Nicolas, SANNIE Thomas) contributed with the communication actions for participants. The members of the FranceCoag registry (Pascal AUQUIER, Karine BAUMSTARCK, Mohamed BOUCEKINE, Hervé CHAMBOST, Vanessa MILIEN, Clémence TABELE, Marie VIPREY, Romain VOLTZENLOGEL) contributed by supplying datasets of the registry. Ngoc Anh Thu NGUYEN, Noémie RESSEGUIER, Marie‐Anaïs ROQUES and Martin POSTZICH performed the analyses. Marie‐Anaïs ROQUES, Noémie RESSEGUIER, Pascal AUQUIER, Hervé CHAMBOST, Ngoc Anh Thu NGUYEN, Any BELTRAN ANZOLA and Martin POSTZICH wrote the draft of the manuscript. All authors critically reviewed the manuscript, provided input on data interpretation, and approval the final version of manuscript.

## Disclosures

The authors stated that they had no interests which might be perceived as posing a conflict or bias.

## Ethics Statement

The study was approved by the French Ethical Research Committee (reference number 16.065), by the French National Agency for the Safety of Medicines and Health Products (reference number ID RCB: 2016‐A01034‐47) and by the French Data Protection Authority (reference number DR‐2018‐060), whose principles are in line with those of the General Data Protection Regulation. The protocol was registered at ClinicalTrials.gov (NCT02866526). To participate in the quantitative phase of this study, written consent was obtained from the YAs and the parents (or legal representatives) of the adolescents. Additional consent for voice recording was obtained from participants who agreed to participate in the qualitative phase of the study.

## Data Availability

The data that support the findings of this study are available on request from the corresponding author. The data are not publicly available due to privacy or ethical restrictions.

## 1 TRANSHEMO Study Group

Coauthors participating in the TRANSHEMO Study Group are listed in alphabetical order: APOSTOLIDIS Thémis (Aix‐Marseille University, LPS / UR 849, Social Psychology Laboratory, Aix‐en‐Provence, France); ARDILLON Laurent (University Hospital of Tours, Haemophilia Treatment Centre, Tours, France); BAUMSTARCK Karine (Aix‐Marseille University, CEReSS / UR 3279 ‐ Center for Studies and Research on Health Services and Quality of Life, Marseille, France); BAYART Sophie (University Hospital of Rennes, Haemophilia Treatment Centre, Rennes, France); BERGER Claire (University Hospital of Saint‐Etienne, Haemophilia Treatment Centre, Saint‐Etienne, France); BERTRAND Marie‐Anne (University Hospital of Besançon, Haemophilia Treatment Centre, Besançon, France); BETSCH Carol (French Association for people with Haemophilia (AFH), Paris, France); BOREL‐DERLON Annie (University Hospital of Caen, Haemophilia Treatment Centre, Caen, France); BOUCEKINE Mohamed (Aix‐Marseille University, CEReSS / UR 3279 ‐ Center for Studies and Research on Health Services and Quality of Life, Marseille, France); CALVEZ Thierry (Sorbonne University, UPMC University of Paris 06, INSERM, UMR‐S 1136, Pierre Louis Institute of Epidemiology and Public Health, Paris, France); CHAMOUNI Pierre (University Hospital of Rouen, Haemophilia Treatment Centre, Rouen, France); CLAEYSSENS DONADEL Ségolène (University Hospital of Toulouse, Haemophilia Treatment Centre, Toulouse, France); COLLE Yannick (French Association for people with Haemophilia (AFH), Paris, France); DARGAUD Yesim (University Hospital of Lyon (HCL), Haemophilia Treatment Centre, Hospital Louis Pradel, Bron, France); de RAUCOURT Emmanuelle (Hospital of Versailles, Haemophilia Treatment Centre, Versailles, France); DESPREZ Dominique (University Regional Hospital of Strasbourg, Haemophilia Treatment Centre, Strasbourg, France); FALAISE Céline (AP‐HM, Haemophilia Treatment Centre, Children Hospital La Timone, Marseille, France); FOURNEL Alexandra (University Hospital of Besançon, Haemophilia Treatment Centre, Besançon, France); FROTSCHER Birgit (University Hospital of Nancy, Haemophilia Treatment Centre, Nancy, France); GAY Valérie (Hospital of Chambéry, Haemophilia Treatment Centre, Chambéry, France); GENRE‐VOLOT Fabienne (University Hospital of Dijon, Haemophilia Treatment Centre, Dijon, France); GOUDEMAND Jenny (University Regional Hospital of Lille, Haemophilia Treatment Centre, Lille, France); GRUEL Yves (University Hospital of Tours, Haemophilia Treatment Centre, Tours, France); GUILLET Benoît (University Hospital of Rennes, Haemophilia Treatment Centre, Rennes, France); HASSOUN Abel (Hospital of Simone Veil d'Eaubonne, Haemophilia Treatment Centre, Montmorency, France); HOCHART Audrey (University Regional Hospital of Lille, Haemophilia Treatment Centre, Lille, France); LAMBERT Thierry (AP‐HP, Haemophilia Treatment Centre, Hospital Bicêtre, Paris, France); LEBRETON Aurélien (University Hospital of Clermont‐Ferrand, Haemophilia Treatment Centre, Clermont‐Ferrand, France); LEROY Tanguy (Aix‐Marseille University, CEReSS / UR 3279 ‐ Center for Studies and Research on Health Services and Quality of Life, Marseille, France); MARLU Raphaël (University Hospital of Grenoble, Haemophilia Treatment Centre, Grenoble, France); MARTIN Michèle (University Hospital of Nancy, Haemophilia Treatment Centre, Nancy, France); MILIEN Vanessa (AP‐HM, Haemophilia Treatment Centre, Children Hospital La Timone, Marseille, France); MONPOUX Fabrice (University Hospital of Nice, Haemophilia Treatment Centre, Nice, France); MORANGE Pierre (Laboratory of Haematology, Hospital La Timone, Marseille, France); MOUREY Guillaume (University Hospital of Besançon, Haemophilia Treatment Centre, Besançon, France); NEGRIER Claude (University Hospital of Lyon (HCL), Haemophilia Treatment Centre, Hospital Louis Pradel, Bron, France); NGUYEN Philippe (University Hospital of Reims, Haemophilia Treatment Centre, Reims, France); NYOMBE Placide (University Hospital of Reunion, Haemophilia Treatment Centre, Reunion Island, France); OUDOT‐CHALLARD Caroline (University Hospital of Limoges, Haemophilia Treatment Centre, Limoges, France); PAN‐PETESCH Brigitte (University Hospital of Brest, Haemophilia Treatment Centre, Brest, France); POLACK Benoît (University Hospital of Grenoble, Haemophilia Treatment Centre, Grenoble, France); RAFOWICZ Anne (Hospital of Versailles, Haemophilia Treatment Centre, Versailles, France / AP‐HP, Haemophilia Treatment Centre, Hospital Bicêtre, Paris, France); RAUCH Antoine (University Regional Hospital of Lille, Haemophilia Treatment Centre, Lille, France); RIVAUD Delphine (University Hospital of Reunion, Haemophilia Treatment Centre, Reunion Island, France); ROHRLICH Pierre‐Simon (University Hospital of Nice, Haemophilia Treatment Centre, Nice, France); SCHNEIDER Pascale (University Hospital of Rouen, Haemophilia Treatment Centre, Rouen, France); SPIEGEL Alexandra (University Regional Hospital of Strasbourg, Haemophilia Treatment Centre, Strasbourg, France); STOVEN Cécile (University Hospital of Reunion, Haemophilia Treatment Centre, Reunion Island, France); SUSEN Sophie (University Regional Hospital of Lille, Haemophilia Treatment Centre, Lille, France); TARDY Brigitte (University Hospital of Saint‐Etienne, Haemophilia Treatment Centre, Saint‐Etienne, France); TROSSAËRT Marc (University Hospital of Nantes, Haemophilia Treatment Centre, Nantes, France); VALENTIN Jean‐Baptiste (University Hospital of Tours, Haemophilia Treatment Centre, Tours, France); VANDERBECKEN Stéphane (University Hospital of Reunion, Haemophilia Treatment Centre, Reunion Island, France); VIPREY Marie (Aix‐Marseille University, CEReSS / UR 3279 ‐ Center for Studies and Research on Health Services and Quality of Life, Marseille, France); VOLTZENLOGEL Romain (AP‐HM, FranceCoag Network, Marseille, France); VOYER‐EBRARD Annelise (University Hospital of Amiens, Haemophilia Treatment Centre, Amiens, France); WIBAUT Bénédicte (University Regional Hospital of Lille, Haemophilia Treatment Centre, Lille, France).

## References

[1] A. Srivastava , E. Santagostino , A. Dougall , et al., “WFH Guidelines for the Management of Hemophilia, 3rd Edition,” Haemophilia: The Official Journal of the World Federation of Hemophilia 26, no. S6 (2020): 1–158. 32744769 10.1111/hae.14046

[2] G. Young , “From Boy to Man: Recommendations for the Transition Process in Haemophilia,” Haemophilia: The Official Journal of the World Federation of Hemophilia 18, no. S5 (2012): 27–32. 22757681 10.1111/j.1365-2516.2012.02893.x

[3] R. W. Blum , D. Garell , C. H. Hodgman , et al., “Transition From Child‐Centered to Adult Health‐Care Systems for Adolescents With Chronic Conditions. A Position Paper of the Society for Adolescent Medicine,” Journal of Adolescent Health: Official Publication of the Society for Adolescent Medicine 14, no. 7 (1993): 570–576. 8312295 10.1016/1054-139x(93)90143-d

[4] R. Crowley , I. Wolfe , K. Lock , et al., “Improving the Transition Between Paediatric and Adult Healthcare: A Systematic Review,” Archives of Disease in Childhood 96, no. 6 (2011): 548–553. 21388969 10.1136/adc.2010.202473

[5] N. Resseguier , N. Rosso‐Delsemme , and A. Beltran Anzola , “Determinants of Adherence and Consequences of the Transition From Adolescence to Adulthood Among Young People With Severe Haemophilia (TRANSHEMO): Study Protocol for a Multicentric French National Observational Cross‐Sectional Study,” BMJ Open 8, no. 7 (2018): e022409. 10.1136/bmjopen-2018-022409 PMC6067371 30049701

[6] American Academy of Pediatrics , American Academy of Family Physicians , American College of Physicians‐American Society of Internal Medicine , “A Consensus Statement on Health Care Transitions for Young Adults With Special Health Care Needs,” Pediatrics 110, no. 6 Pt 2 (2002): 1304–1306. 12456949

[7] D. S. Rosen , R. W. Blum , M. Britto , et al., “Transition to Adult Health Care for Adolescents and Young Adults With Chronic Conditions: Position Paper of the Society for Adolescent Medicine,” Journal of Adolescent Health: Official Publication of the Society for Adolescent Medicine 33, no. 4 (2003): 309–311. 14519573 10.1016/s1054-139x(03)00208-8

[8] P. Moons , D. Hilderson , and K. Van Deyk , “Implementation of Transition Programs Can Prevent Another Lost Generation of Patients With Congenital Heart Disease,” European Journal of Cardiovascular Nursing 7, no. 4 (2008): 259–263. 19013410 10.1016/j.ejcnurse.2008.10.001

[9] A. R. Watson , “Non‐Compliance and Transfer From Paediatric to Adult Transplant Unit,” Pediatric Nephrology 14, no. 6 (2000): 469–472. 10872185 10.1007/s004670050794

[10] L. A. Schwartz , L. K. Tuchman , W. L. Hobbie , et al., “A Social‐Ecological Model of Readiness for Transition to Adult‐Oriented Care for Adolescents and Young Adults With Chronic Health Conditions,” Child Care Health and Development 37, no. 6 (2011): 883–895. 22007989 10.1111/j.1365-2214.2011.01282.x

[11] S. E. McLaughlin , M. Diener‐West , A. Indurkhya , et al., “Improving Transition From Pediatric to Adult Cystic Fibrosis Care: Lessons From a National Survey of Current Practices,” Pediatrics 121, no. 5 (2008): e1160–e1166. 18450860 10.1542/peds.2007-2217

[12] H W. Parker , “Transition and Transfer of Patients Who Have Cystic Fibrosis to Adult Care,” Clinics in Chest Medicine 28, no. 2 (2007): 423–432. 17467557 10.1016/j.ccm.2007.02.007

[13] D. Allen and J. Gregory , “The Transition From Children's to Adult Diabetes Services: Understanding the 'Problem',” Diabetic Medicine: A Journal of the British Diabetic Association 26, no. 2 (2009): 162–166. 19236619 10.1111/j.1464-5491.2008.02647.x

[14] F. Cadario , F. Prodam , S. Bellone , et al., “Transition Process of Patients With Type 1 Diabetes (T1DM) From Paediatric to the Adult Health Care Service: A Hospital‐Based Approach,” Clinical Endocrinology 71, no. 3 (2009): 346–350. 19178523 10.1111/j.1365-2265.2008.03467.x

[15] P. Owen and D. Beskine , “Factors Affecting Transition of Young People With Diabetes,” Paediatric Nursing 20, no. 7 (2008): 33–38. 10.7748/paed2008.09.20.7.33.c6706 18808056

[16] M. McPherson , L. Thaniel , and C P. Minniti , “Transition of Patients With Sickle Cell Disease From Pediatric to Adult Care: Assessing Patient Readiness,” Pediatric Blood & Cancer 52, no. 7 (2009): 838–841. 19229973 10.1002/pbc.21974

[17] D. V. Schidlow and S B. Fiel , “Life Beyond Pediatrics. Transition of Chronically Ill Adolescents From Pediatric to Adult Health Care Systems,” Medical Clinics of North America 74, no. 5 (1990): 1113–1120. 2201847 10.1016/s0025-7125(16)30505-3

[18] P. K. Davis , A. Young , H. Cherry , et al., “Increasing the Happiness of Individuals With Profound Multiple Disabilities: Replication and Extension,” Journal of Applied Behavior Analysis 37, no. 4 (2004): 531–534. 15669414 10.1901/jaba.2004.37-531 PMC1284532

[19] L. Granek , P. C. Nathan , Z. R. S. Rosenberg‐Yunger , et al., “Psychological Factors Impacting Transition From Paediatric to Adult Care by Childhood Cancer Survivors,” Journal of Cancer Survivorship: Research and Practice 6, no. 3 (2012): 260–269. 22547096 10.1007/s11764-012-0223-0

[20] A. L. van Staa , S. Jedeloo , J. van Meeteren , et al., “Crossing the Transition Chasm: Experiences and Recommendations for Improving Transitional Care of Young Adults, Parents and Providers,” Child Care Health and Development 37, no. 6 (2011): 821–832. 22007982 10.1111/j.1365-2214.2011.01261.x

[21] P. Scal , “Transition for Youth With Chronic Conditions: Primary Care Physicians' Approaches,” Pediatrics 110, no. 6 Pt 2 (2002): 1315–1321. 12456951

[22] N. G. Peter , C. M. Forke , K. R. Ginsburg , et al., “Transition From Pediatric to Adult Care: Internists' perspectives,” Pediatrics 123, no. 2 (2009): 417–423. 19171604 10.1542/peds.2008-0740

[23] J. St‐Louis , P. Chowdary , G. Dolan , et al., “Multidisciplinary Team Care of Patients With Hemophilic Arthropathy: A Qualitative Assessment of Contemporary Practice in the UK and Canada : Canada/UK: MDT Practices for Hemophilia,” Clinical and Applied Thrombosis/Hemostasis: Official Journal of the International Academy of Clinical and Applied Thrombosis/Hemostasis 28 (2022): 10760296211070002. 35060765 10.1177/10760296211070002 PMC8796082

[24] V. R. Breakey , V. Bouskill , C. Nguyen , et al., “Online Peer‐to‐Peer Mentoring Support for Youth With Hemophilia: Qualitative Needs Assessment,” JMIR Pediatrics and Parenting 1, no. 2 (2018): e10958. 31518296 10.2196/10958 PMC6715049

[25] J. W. Hoefnagels , M. C. Kars , K. Fischer , et al., “The Perspectives of Adolescents and Young Adults on Adherence to Prophylaxis in Hemophilia: A Qualitative Study,” Patient Prefer Adherence 14 (2020): 163–171. 32158199 10.2147/PPA.S232393 PMC6986248

[26] M. Witkop , C. Guelcher , A. Forsyth , et al., “Challenges in Transition to Adulthood for Young Adult Patients With Hemophilia: Quantifying the Psychosocial Issues and Developing Solutions,” American Journal of Hematology 90, no. S2 (2015): S1–S2. 10.1002/ajh.24217 26619191

[27] K. Beny , S. du , B. de Vigneulles , et al., “Patients' Perception of the Impact of Innovation on Hemophilia Care Management Organization: A Qualitative Study Protocol (INNOVHEMO Study),” Patient Prefer Adherence 15 (2021): 1807–1815. 34434044 10.2147/PPA.S322531 PMC8380624

[28] N. A. T. Nguyen , and P. Auquier , and A. Beltran Anzola , “Determinants of Adherence and Consequences of the Transition From Adolescence to Adulthood Among Young People With Severe Haemophilia (TRANSHEMO): A Multicentric French National Observational Cross‐Sectional Study Based on the FranceCoag Registry,” Haemophilia: The Official Journal of the World Federation of Hemophilia 29, no. 5 (2023): 1202–1218. 37572328 10.1111/hae.14841

[29] V. Braun and V. Clarke , “Using Thematic Analysis in Psychology,” Qualitative Research in Psychology 3 (2006): 77–101.

[30] L. H. Schrijvers , M. C. Kars , M. Beijlevelt‐van der Zande , et al., “Unravelling Adherence to Prophylaxis in Haemophilia: A Patients' Perspective,” Haemophilia: The Official Journal of the World Federation of Hemophilia 21, no. 5 (2015): 612–621. 25858411 10.1111/hae.12660

[31] B. Polack , T. Calvez , H. Chambost , et al., “EQOFIX: A Combined Economic and Quality‐of‐Life Study of Hemophilia B Treatments in France,” Transfusion 55, no. 7 (2015): 1787–1797. 25652955 10.1111/trf.13016

[32] S. B. van Os , N. A. Troop , K. R. Sullivan , et al., “Adherence to Prophylaxis in Adolescents and Young Adults With Severe Haemophilia: A Quantitative Study With Patients,” PLoS ONE 12, no. 1 (2017): e0169880. 28103266 10.1371/journal.pone.0169880 PMC5245860

[33] S. J. Lane , I. Walker , A. K. Chan , et al., “Treatment Decision‐Making Among Canadian Youth With Severe Haemophilia: A Qualitative Approach,” Haemophilia: The Official Journal of the World Federation of Hemophilia 21, no. 2 (2015): 180–189. 25296666 10.1111/hae.12543

[34] K. Khair , F. Gibson , and L. Meerabeau , “The Benefits of Prophylaxis: Views of Adolescents With Severe Haemophilia,” Haemophilia: The Official Journal of the World Federation of Hemophilia 18, no. 3 (2012): e286–e289. 21910789 10.1111/j.1365-2516.2011.02644.x

[35] L. Wintz , T. Sannié , S. Ayçaguer , et al., “Patient Resources in the Therapeutic Education of Haemophiliacs in France: Their Skills and Roles as Defined by Consensus of a Working Group,” Haemophilia: The Official Journal of the World Federation of Hemophilia 16, no. 3 (2010): 447–454. 20088955 10.1111/j.1365-2516.2009.02163.x

[36] J. M. McLaughlin , M. L. Witkop , A. Lambing , et al., “Better Adherence to Prescribed Treatment Regimen Is Related to Less Chronic Pain Among Adolescents and Young Adults With Moderate or Severe Haemophilia,” Haemophilia: The Official Journal of the World Federation of Hemophilia 20, no. 4 (2014): 506–512. 24517097 10.1111/hae.12360

[37] M. García‐Dasí , J. A. Aznar , V. Jiménez‐Yuste , et al., “Adherence to Prophylaxis and Quality of Life in Children and Adolescents With Severe Haemophilia A,” Haemophilia: The Official Journal of the World Federation of Hemophilia 21, no. 4 (2015): 458–464. 25649244 10.1111/hae.12618

[38] P. De Moerloose , W. Urbancik , and H. M. Van Den Berg , “A Survey of Adherence to Haemophilia Therapy in Six European Countries: Results and Recommendations,” Haemophilia: The Official Journal of the World Federation of Hemophilia 14, no. 5 (2008): 931–938. 18684125 10.1111/j.1365-2516.2008.01843.x

[39] C. Fair , J. Cuttance , N. Sharma , et al., “International and Interdisciplinary Identification of Health Care Transition Outcomes,” JAMA Pediatrics 170, no. 3 (2016): 205–211. 26619178 10.1001/jamapediatrics.2015.3168 PMC6345570

[40] N. Bhatt , L. Boggio , and M L. Simpson , “Using an Educational Intervention to Assess and Improve Disease‐Specific Knowledge and Health Literacy and Numeracy in Adolescents and Young Adults With Haemophilia A and B,” Haemophilia: The Official Journal of the World Federation of Hemophilia 27, no. 2 (2021): 229–236. 33590938 10.1111/hae.14228

